# Food Allergen Component Sensitization Patterns in Eosinophilic Esophagitis: Insights from a Retrospective Comparative Study

**DOI:** 10.3390/foods15040748

**Published:** 2026-02-18

**Authors:** Adam Wawrzeńczyk, Katarzyna Napiórkowska-Baran, Kinga Lis, Marta Tykwińska, Maciej Szota, Paweł Treichel, Justyna Durślewicz, Zbigniew Bartuzi

**Affiliations:** 1 Department of Allergology, Clinical Immunology and Internal Diseases, Collegium Medicum in Bydgoszcz, Nicolaus Copernicus University in Toruń, 85-067 Bydgoszcz, Poland; knapiorkowska@cm.umk.pl (K.N.-B.); kinga.lis@cm.umk.pl (K.L.); marta.tykwinska@cm.umk.pl (M.T.); maciejszota98@gmail.com (M.S.); zbartuzi@cm.umk.pl (Z.B.); 2 Student Research Club of Clinical Immunology, Department of Allergology, Clinical Immunology and Internal Diseases, Collegium Medicum in Bydgoszcz, Nicolaus Copernicus University in Toruń, 85-067 Bydgoszcz, Poland; treichel.pawel@gmail.com; 3 Faculty of Medicine, Bydgoszcz University of Science and Technology, Aleje Prof. S. Kaliskiego 7, 85-796 Bydgoszcz, Poland; justyna.durslewicz@pbs.edu.pl; 4 Department of Tumor Pathology and Pathomorphology, Oncology Centre—Prof. Franciszek Łukaszczyk Memorial Hospital, 85-796 Bydgoszcz, Poland

**Keywords:** eosinophilic esophagitis, food allergy, food allergen components, component-resolved diagnostics, lipid transfer proteins, food allergen stability, elimination diet

## Abstract

Eosinophilic esophagitis (EoE) is a chronic, food-driven inflammatory disorder of the esophagus in which repeated exposure to dietary antigens plays a central role, yet identification of clinically relevant food triggers remains largely empirical. In this retrospective, single-center study, molecular IgE sensitization profiles were descriptively characterized in adult patients with EoE (*n* = 22) and compared with an allergic control group with chronic urticaria (CU; *n* = 29) using component-resolved diagnostics. IgE sensitization was common in both cohorts and predominantly reflected inhalant-related, cross-reactive components, particularly PR-10 proteins (63.6% in EoE vs. 37.9% in CU). In contrast, sensitization to structurally stable food allergen components, including lipid transfer proteins and plant storage proteins, was observed in a subset of patients with EoE (31.8%) and was not detected in the control group (0%; *p* = 0.0015). These food-derived components are characterized by resistance to thermal processing and gastrointestinal digestion and may reflect patterns of sustained dietary exposure rather than acute IgE-mediated reactions. Consistent with previous observations, component-resolved diagnostics showed limited utility for the direct identification of trigger foods in eosinophilic esophagitis. Accordingly, the observed molecular sensitization patterns should be interpreted as descriptive and hypothesis-generating signals rather than as indicators of pathogenic mechanisms or clinical decision-making tools. The findings highlight the importance of considering molecular properties of food allergen components when interpreting sensitization profiles in chronic, non-IgE-mediated inflammatory diseases and underscore the need for prospective studies integrating standardized clinical and dietary outcomes.

## 1. Introduction

### 1.1. Eosinophilic Esophagitis as a Food-Driven Allergic Disease

Eosinophilic esophagitis (EoE) is a chronic, immune-mediated inflammatory disorder of the esophagus characterized by eosinophil-predominant infiltration and a strong association with atopic disease. Although EoE frequently coexists with IgE-mediated food allergy, accumulating clinical evidence indicates that it represents a distinct, food-driven condition in which dietary antigens play a central role in disease initiation and persistence [1].

The causal role of food exposure in EoE is best supported by interventional dietary studies. Empiric elimination diets targeting common food triggers induce histologic remission in approximately 50–75% of adult patients, while elemental amino acid-based diets achieve even higher response rates [2]. Systematic food reintroduction consistently results in recurrence of esophageal eosinophilia and clinical symptoms, demonstrating that specific dietary components actively drive disease activity rather than representing epiphenomena of inflammation [2,3]. Long-term follow-up studies further show that sustained avoidance of identified trigger foods can maintain histologic and clinical remission, underscoring the importance of continuous antigen exposure in disease maintenance [3].

Despite this clear food dependence, conventional allergy diagnostics have limited utility in identifying causative foods in EoE. Skin prick testing and serum food-specific IgE measurements show poor concordance with elimination–reintroduction outcomes, predicting only a minority of clinically relevant triggers [2]. These observations indicate that classical IgE-mediated mechanisms are not the primary drivers of esophageal inflammation in most patients with EoE.

Mechanistic studies further support this distinction, demonstrating that EoE is predominantly mediated by non-IgE pathways. Interventional trials targeting IgE have failed to improve histologic or clinical outcomes, while tissue-based analyses reveal enrichment of IgG4-positive plasma cells and evidence of antigen-specific T-cell activation within the esophageal mucosa [4,5]. Accordingly, IgE sensitization in eosinophilic esophagitis should be viewed primarily as a marker of antigen exposure and immunological imprinting rather than as a direct effector mechanism of disease.

### 1.2. Molecular Properties of Food Allergens as Food Ingredients: Stability, Structure, and Clinical Relevance

In this manuscript, the term “structurally stable food allergen components” refers to allergenic proteins characterized by intrinsic biochemical properties that confer resistance to thermal processing and gastrointestinal digestion. These properties include compact tertiary structure, stabilizing intramolecular interactions such as disulfide bonds, and high conformational stability, allowing such proteins to remain immunologically intact during food processing and gastrointestinal transit. Representative examples include non-specific lipid transfer proteins and plant seed storage proteins.

In food allergy, the clinically relevant unit is rarely the “food” itself but rather specific proteins—food ingredients—whose molecular properties determine stability during processing and digestion, epitope presentation, and immune recognition. Across major allergen families, resistance to thermal denaturation and gastrointestinal proteolysis is a recurring feature of class I food allergens, which can sensitize via the gastrointestinal tract and are more frequently associated with systemic reactions [6,7]. In contrast, class II pollen-related food allergens, including PR-10 proteins and profilins, are typically heat-labile and digestion-sensitive, yet remain clinically relevant through primary inhalant sensitization and subsequent IgE cross-reactivity with homologous food proteins [6].

Several allergen families illustrate how molecular structure and stability influence clinical expression. Plant non-specific lipid transfer proteins (nsLTPs) are consistently described as highly resistant to heat and proteolysis, supporting their persistence despite culinary processing and gastrointestinal conditions [6,8]. Similarly, plant seed storage proteins derive stability from compact folding and stabilizing intramolecular interactions, facilitating survival during digestion [6,7]. Peanut storage proteins exemplify this paradigm, as resistance to thermal stress and proteolytic degradation does not necessarily translate into reduced allergenic potency [9].

Beyond plant-derived proteins, molecular diagnostic panels include stable animal allergens such as shellfish tropomyosin and fish parvalbumin, which likewise resist processing and digestion [8,10]. Importantly, food processing does not uniformly reduce allergenicity. While extensive heating may destroy conformational epitopes in labile allergens, processing-induced modifications such as glycation during high-temperature treatment may enhance immunogenicity in selected contexts [8,10]. Gastric digestion further acts as a physiological gatekeeper, and impaired digestion has been associated with de novo IgE formation to dietary proteins, underscoring host-related modifiers of sensitization independent of the food ingredient itself [11]. Collectively, these observations highlight the relevance of molecular stability when interpreting sensitization profiles in a food-science context [12].

### 1.3. Panallergens and Cross-Reactivity Patterns in Food Allergy

Panallergens are structurally conserved protein families shared across phylogenetically unrelated allergen sources and constitute the molecular basis of IgE cross-reactivity between inhalant and food allergens [13,14]. Clinically relevant panallergen families include lipid transfer proteins (LTPs), PR-10 proteins, profilins, tropomyosins, and parvalbumins, each characterized by distinct biochemical properties and sensitization pathways.

Among these, lipid transfer proteins represent a clinically important group due to their resistance to thermal processing, proteolytic digestion, and acidic gastric conditions. This intrinsic stability supports broad IgE cross-reactivity across botanically unrelated plant foods and is associated with systemic reactions rather than localized symptoms [15,16,17].

In contrast, PR-10 proteins and profilins are heat-labile and readily degraded during digestion. Sensitization typically arises from primary inhalant exposure, most commonly birch pollen, with subsequent IgE cross-reactivity to homologous food proteins. Although such sensitization often results in multiple positive molecular test results, clinical manifestations are usually mild and confined to oral allergy syndrome [13,18], and PR-10 proteins account for the majority of cross-reactive sensitizations detected in adults with combined inhalant and food allergy [18,19].

A key diagnostic challenge associated with panallergen sensitization is the frequent dissociation between IgE positivity and clinically relevant food allergy. High rates of sensitization to broadly cross-reactive components often exceed rates of confirmed food allergy, highlighting the limitations of conventional diagnostics and the risk of unnecessary dietary restriction [16,20]. In eosinophilic esophagitis, where chronic tissue-restricted inflammation is not directly driven by IgE effector pathways, panallergen sensitization is therefore best interpreted as a marker of background cross-reactivity rather than as an indicator of causative food triggers [21,22].

### 1.4. Molecular Diagnostics in Food Allergy and the Unmet Need in Eosinophilic Esophagitis

Conventional extract-based allergy diagnostics remain widely used in clinical practice but are limited by poor specificity and an inability to distinguish clinically relevant food allergy from asymptomatic sensitization, particularly in polysensitized individuals [23]. Population-based studies demonstrate that only a minority of sensitized patients develop clinically confirmed food allergy upon oral food challenge, highlighting the risk of overdiagnosis when extract-based testing is used in isolation [24].

Component-resolved diagnostics (CRD) address some of these limitations by enabling IgE detection against individual allergenic molecules. Across multiple food allergens, CRD has improved diagnostic accuracy through identification of stable components associated with systemic reactions and through differentiation between primary food sensitization and pollen-related cross-reactivity [23,25].

Despite these advantages, the role of CRD in eosinophilic esophagitis remains insufficiently defined. Molecular profiling studies consistently demonstrate high rates of IgE sensitization in EoE—predominantly to aeroallergens and cross-reactive plant components such as PR-10 proteins and profilins—yet these patterns correlate poorly with food triggers identified by elimination diets and histologic response [26]. Currently available CRD platforms were largely developed to predict IgE-mediated anaphylaxis rather than chronic, non-IgE-driven esophageal inflammation, and allergenic components relevant to major EoE trigger foods remain incompletely characterized [27].

Accordingly, this study aimed to characterize component-resolved IgE sensitization patterns in adults with eosinophilic esophagitis and to compare these profiles with those observed in a clinically relevant comparator group with chronic urticaria. By focusing on sensitization to structurally stable food allergen components in contrast to predominantly inhalant-driven and cross-reactive patterns, we sought to provide a food ingredient-centered framework for interpreting molecular IgE profiles in EoE.

**Figure 1:**
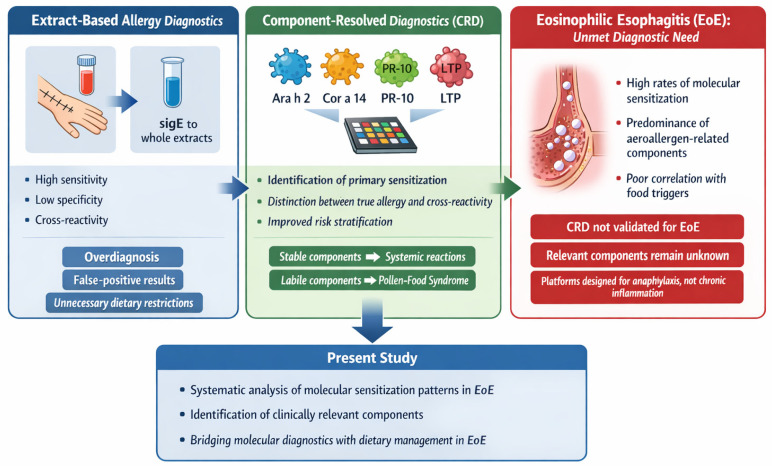
Conceptual framework illustrating the relationship between extract-based allergy diagnostics, component-resolved diagnostics (CRD), and the diagnostic gap in eosinophilic esophagitis (EoE). Extract-based testing is characterized by high sensitivity but limited specificity due to cross-reactivity. CRD enables identification of sensitization to individual allergenic molecules and differentiation between primary food allergy and pollen-related cross-reactivity. In EoE, molecular sensitization profiles frequently show limited concordance with food triggers identified by elimination diets, underscoring a diagnostic gap in the context of chronic, non-IgE-driven esophageal inflammation. The figure was created by the authors for illustrative purposes using standard vector graphic software and does not represent data-derived, mechanistic, or causal relationships; no AI-assisted tools were used in its preparation.

The conceptual relationship between extract-based diagnostics, CRD, and the unresolved diagnostic gap in eosinophilic esophagitis is summarized in Figure 1.

## 2. Materials and Methods

### 2.1. Study Design and Ethical Considerations

This study was designed as a retrospective, observational analysis based on a review of medical records from adult patients evaluated at the Department of Allergology, Clinical Immunology and Internal Diseases, Dr. Jan Biziel University Hospital No. 2 in Bydgoszcz, Poland, between 2012 and 2025.

The study was non-interventional in nature and did not involve any additional diagnostic or therapeutic procedures, direct patient contact, or modifications of routine clinical management. All data were obtained exclusively from existing medical documentation, including clinical information, laboratory findings, and results of component-resolved IgE allergy diagnostics.

Data analysis was conducted using anonymized and coded patient records to ensure confidentiality and full compliance with applicable data protection regulations. The study protocol was approved by the Bioethics Committee of the Faculty of Medicine, Collegium Medicum in Bydgoszcz, Nicolaus Copernicus University in Toruń (approval no. KEWL 35/2025, issued on 17 December 2025) in accordance with national regulations, the General Data Protection Regulation (GDPR), and the principles of the Declaration of Helsinki.

### 2.2. Study Objectives and Hypothesis

The primary objective of this study was to characterize molecular IgE sensitization patterns—using component-resolved IgE diagnostics—in adult patients with eosinophilic esophagitis (EoE) diagnosed in accordance with the American College of Gastroenterology (ACG) clinical guideline, and to compare these patterns with those observed in a control group of patients with chronic urticaria (CU) diagnosed according to the international EAACI/GA^2^LEN/EuroGuiDerm/APAAACI urticaria guideline.

Specifically, the analysis aimed to explore differences in the prevalence and distribution of molecular IgE sensitization to food- and inhalant-derived allergen components, with particular emphasis on allergen groups differing in molecular stability and clinical relevance. The study further sought to examine potential differences between the groups in sensitization to structurally stable food allergens, such as lipid transfer proteins and plant storage proteins, compared with predominantly inhalant-related or cross-reactive allergen components.

An additional objective was to assess the coexistence of other allergic diseases and selected clinical characteristics in patients with EoE and CU in order to provide broader phenotypic context for the observed sensitization patterns.

Given the retrospective and exploratory nature of the study, we hypothesized that molecular IgE sensitization profiles in EoE differ from those observed in CU, with a potential enrichment of sensitization to structurally stable food allergen components. These analyses were intended to be hypothesis-generating rather than confirmatory and were not designed to establish causality or identify specific trigger foods.

### 2.3. Study Population and Eligibility Criteria

The study population consisted of adult patients diagnosed with eosinophilic esophagitis (EoE) and a control group of patients with chronic urticaria (CU) who were hospitalized or evaluated at the study center during the defined study period.

Eligible participants were adults aged 18–75 years. Inclusion criteria comprised a documented diagnosis of eosinophilic esophagitis or chronic urticaria and the availability of molecular allergy testing results. Patients with incomplete clinical records or missing component-resolved diagnostic data were excluded from the analysis.

Eosinophilic esophagitis was diagnosed based on symptoms of esophageal dysfunction and histological confirmation of eosinophilic inflammation (≥15 eosinophils per high-power field) in esophageal biopsies, with secondary causes of esophageal eosinophilia excluded, as documented in the medical records. Chronic urticaria was defined as recurrent wheals, angioedema, or both persisting for >6 weeks, and diagnosed by an allergy specialist according to current guideline-based criteria.

Patients with chronic urticaria (CU) were selected as a pragmatic comparator group from the same tertiary allergy setting, where component-resolved IgE testing was performed as part of routine clinical evaluation. Because CU does not involve eosinophilic gastrointestinal inflammation and is generally not a food-driven mucosal disease, it provides a clinically relevant reference for background IgE sensitization and cross-reactivity when interpreting molecular sensitization patterns observed in EoE.

Because the comparator group was not matched for age, sex, or atopic status and no multivariable adjustment was performed, between-group differences should be interpreted cautiously as descriptive associations that may be partly influenced by demographic or atopy-related confounding.

### 2.4. Molecular Allergy Diagnostics

Molecular allergy diagnostics were performed using component-resolved IgE testing (CRD) as part of routine clinical practice. For the purposes of this study, results obtained during standard diagnostic evaluation were retrospectively analyzed. No additional laboratory testing was performed specifically for this study.

Two multiplex platforms were used for component-resolved diagnostics: the ImmunoCAP ISAC microarray (Thermo Fisher Scientific, Uppsala, Sweden) and the ALEX2 Allergy Explorer system (Macro Array Diagnostics, Vienna, Austria). Both platforms enable simultaneous measurement of specific IgE antibodies against a broad panel of purified natural and recombinant allergen components using a single serum sample.

The ISAC platform assesses IgE reactivity to more than 100 allergen molecules, including food allergens, inhalant allergens, and cross-reactive panallergens, with results reported semi-quantitatively as ISAC Standardized Units (ISU-E). The ALEX2 system includes a comparable and partially overlapping panel of allergen components and reports quantitative results expressed in kUA/L, while additionally incorporating CCD inhibition to reduce false-positive results related to cross-reactive carbohydrate determinants. Because the ISAC and ALEX2 panels only partially overlap, platform-related differences were considered a potential source of measurement heterogeneity; however, the distribution of diagnostic platforms did not differ between study groups (Table 1). Accordingly, component-level analyses were interpreted descriptively, and no attempt was made to directly compare or pool quantitative IgE values across platforms.

The molecular allergen panels covered major food allergens, including storage proteins (2S albumins, 7S and 11S globulins), lipid transfer proteins, and milk and egg components, as well as panallergens (profilins, PR-10 proteins, tropomyosins) and inhalant allergens (pollens, house dust mites, animal dander, and molds). This broad coverage enabled comprehensive characterization of individual IgE sensitization profiles.

Interpretation of molecular IgE results was based on manufacturer-recommended cut-off values. For ISAC, values ≥ 0.3 ISU-E were considered positive, whereas for ALEX2, specific IgE concentrations ≥ 0.35 kUA/L were regarded as indicative of sensitization. All results were interpreted descriptively for research purposes and were not reclassified or reinterpreted beyond the original clinical laboratory reports.

For the purposes of this study, molecular IgE sensitization profiles were analyzed as immunological markers of exposure and sensitization patterns and were not interpreted as indicators of pathogenic mechanisms.

All laboratory analyses were conducted in certified diagnostic laboratories in accordance with quality control standards applicable at the time of testing. As this was a retrospective observational study, molecular sensitization patterns were analyzed as immunological markers of IgE reactivity and were not equated with clinically confirmed food allergy. Oral food challenges were not systematically performed and were not required for inclusion in the study.

### 2.5. Statistical Analysis

Statistical analyses were performed to describe and compare molecular sensitization patterns and selected clinical characteristics between patients with eosinophilic esophagitis (EoE) and the control group with chronic urticaria (CU).

Peripheral blood eosinophilia was defined as an absolute eosinophil count ≥ 0.55 × 10^9^/L (equivalent to 0.55 G/L) (i.e., above the upper limit of normal reported by the local laboratory) and analyzed both as a binary variable (present vs. absent) and as a continuous measure.

Continuous variables were assessed for normality of distribution using the Shapiro–Wilk test. Normally distributed data are presented as means with standard deviations, whereas non-normally distributed variables are reported as medians with interquartile ranges. Categorical variables are presented as absolute numbers and percentages.

Comparisons between the EoE and CU groups were conducted using Student’s *t*-test for normally distributed continuous variables and the Mann–Whitney U test for non-normally distributed data. Categorical variables were compared using the chi-square test or Fisher’s exact test, as appropriate.

The primary analyses focused on differences in the prevalence of IgE sensitization to selected molecular allergen groups, including food-derived components (such as storage proteins, lipid transfer proteins, and PR-10 proteins), panallergens (profilins and tropomyosins), and inhalant allergens. Secondary analyses explored associations between molecular sensitization profiles and selected clinical features, including the presence of other allergic diseases. Additionally, sensitization to structurally stable food allergen components was also analyzed as a composite endpoint (any LTP or plant storage protein), and exploratory subgroup comparisons were performed within the EoE cohort. Sensitivity analyses stratified by CRD platform (ISAC vs. ALEX2) were also performed for the stable-component endpoint.

Given the exploratory nature of the study and the limited sample size, no formal sample size calculation was performed. All analyses were considered descriptive and hypothesis-generating. A two-sided *p*-value < 0.05 was considered statistically significant.

Statistical analyses were performed using Statistica (version 13.3; TIBCO Software Inc., Palo Alto, CA, USA).

Given the retrospective design, limited sample size, and the rarity of eosinophilic esophagitis, all statistical analyses were intended to be descriptive and hypothesis-generating rather than confirmatory.

No correction for multiple comparisons was applied due to the exploratory nature of the study; therefore, all reported *p*-values should be interpreted as descriptive and hypothesis-generating rather than confirmatory.

No multivariable modeling was performed due to the limited sample size and exploratory design; therefore, potential confounding by age, sex, and atopic background cannot be excluded. Because of partial non-overlap between CRD platforms, component-level findings were evaluated qualitatively rather than as platform-independent estimates.

## 3. Results

### 3.1. Baseline Demographic Characteristics and Type of Molecular Allergy Testing

A total of 22 patients with eosinophilic esophagitis (EoE) and 29 patients with chronic urticaria (CU) were included in the analysis. Patients with EoE were younger than those with CU, with a median age of 28 years (IQR: 23–35) compared with 39 years (IQR: 29–47) in the CU group (*p* = 0.001, Mann–Whitney U test).

Male sex was more frequent in the EoE group than in the CU group (59.1% vs. 31.0%); however, this difference did not reach statistical significance (*p* = 0.053, Fisher’s exact test).

With respect to component-resolved molecular allergy diagnostics, the distribution of diagnostic platforms was comparable between groups. In the EoE cohort, 59.1% of patients were tested using the ImmunoCAP ISAC platform and 40.9% using the ALEX2 system, compared with 58.6% and 41.4%, respectively, in the CU group (*p* = 1.000, Fisher’s exact test).

These demographic differences reflect known epidemiological patterns of eosinophilic esophagitis but may also contribute to differences in sensitization profiles and should therefore be considered a potential source of confounding in between-group comparisons. Baseline demographic characteristics and diagnostic methods are summarized in Table 1.

### 3.2. Clinical Presentation of Eosinophilic Esophagitis

**Figure 2:**
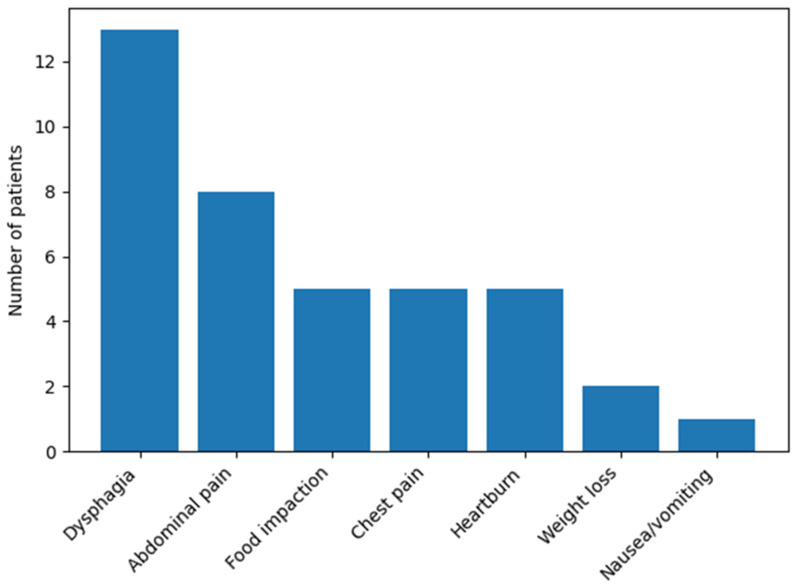
Clinical symptoms reported by patients with eosinophilic esophagitis (*n* = 22). Bars represent the number of patients reporting each symptom. Individual patients could report more than one symptom.

The distribution of self-reported clinical symptoms among patients with eosinophilic esophagitis is shown in Figure 2. Dysphagia was the most commonly reported symptom, affecting 13 of 22 patients, followed by abdominal pain reported by 8 patients. A history of food impaction, retrosternal chest pain, and heartburn was each documented in 5 patients.

Less frequent manifestations included unintentional weight loss, reported by 2 patients, and nausea or vomiting, which was reported by 1 patient. Overall, the symptom profile was dominated by esophageal manifestations, with relatively infrequent systemic or alarm symptoms.

### 3.3. Other Atopic Comorbidities

The prevalence of atopic comorbidities other than eosinophilic esophagitis and chronic urticaria is summarized in Table 2. Allergic rhinitis was reported in 45.5% of patients with eosinophilic esophagitis (EoE) and in 27.6% of patients with chronic urticaria (CU), with no statistically significant difference between groups (*p* = 0.140).

Angioedema was more frequently observed in the CU group than in the EoE group (58.6% vs. 9.1%, *p* < 0.001). The prevalence of asthma did not differ significantly between patients with EoE (36.4%) and those with CU (24.1%, *p* = 0.240).

Atopic dermatitis was infrequent in both cohorts and was documented in one patient with EoE (4.5%) and in none of the patients with CU (*p* = 0.430). A history of anaphylaxis was rare and occurred with comparable frequency in the two groups (9.1% vs. 6.9%, *p* = 1.000).

### 3.4. Overall Detection of Molecular Allergen Components

The overall detection of molecular allergen components is summarized in Table 3. At least one molecular component was detected in 77.3% of patients with eosinophilic esophagitis (EoE) and in 65.5% of patients with chronic urticaria (CU), with no statistically significant difference between groups (*p* = 0.536, Fisher’s exact test).

The median number of detected molecular components was 12.5 (IQR: 2–23) in the EoE group and 6 (IQR: 0–18) in the CU group. This difference did not reach statistical significance (*p* = 0.170, Mann–Whitney U test). A wide interindividual variability in the number of detected components was observed in both cohorts.

### 3.5. Sensitization to Panallergen Families and Selected Component Groups

Sensitization to panallergen families and selected molecular component groups is summarized in Table 4.

Among plant-derived panallergens, PR-10 proteins represented the most frequently detected sensitization pattern in both cohorts. PR-10 sensitization was observed in 63.6% of patients with eosinophilic esophagitis (EoE) and in 37.9% of patients with chronic urticaria (CU); however, this difference did not reach statistical significance (*p* = 0.093). This finding indicates a broadly shared cross-reactive sensitization background rather than a disease-specific pattern.

Sensitization to lipid transfer proteins (LTPs) was detected in a subset of patients with EoE (18.2%) and was not observed in the CU group (*p* = 0.029). Sensitization to plant storage proteins was also detected only in the EoE cohort (13.6%), although this difference did not reach statistical significance (*p* = 0.074). When structurally stable food allergen components were analyzed as a composite endpoint (any sensitization to LTPs or plant storage proteins), sensitization was present in 7 of 22 patients with EoE (31.8%) and in none of the CU controls (0 of 29), yielding a statistically significant difference (*p* = 0.0015).

In platform-stratified sensitivity analyses, sensitization to structurally stable food allergen components remained restricted to patients with EoE in both diagnostic subsets, although subgroup sizes were small and statistical significance was not uniformly retained. Within the EoE cohort, patients sensitized to stable food allergen components exhibited a higher number of detected allergen components overall compared with non-sensitized patients; however, no differences were observed with respect to age, sex distribution, or peripheral blood eosinophilia. Given the limited overlap of individual allergen components between platforms, these findings should be interpreted at the level of allergen families rather than individual molecules.

Sensitization to other panallergen families, including profilins and tropomyosins, was infrequent in both groups and did not differ significantly between cohorts. Animal-derived allergen components corresponding to classical six-food elimination diet items, such as fish parvalbumins, shellfish tropomyosins, and milk-derived serum albumins, were rare and showed no significant between-group differences.

Given the limited sample size and exploratory nature of the analyses, all component-level differences reported in this section should be interpreted descriptively and not as statistically definitive evidence of disease-specific associations.

### 3.6. Molecular Sensitization Patterns to Inhalant- and Food-Related Components

Comparative analysis of component-resolved IgE profiles revealed differences in the prevalence of selected inhalant- and food-related allergen components between patients with eosinophilic esophagitis (EoE) and those with chronic urticaria (CU).

Among aeroallergen-derived components, sensitization to rBet v 1 was more frequently detected in patients with EoE than in CU controls (63.6% vs. 27.6%, *p* = 0.021). Similarly, sensitization to rPhl p 5 (31.8% vs. 6.9%, *p* = 0.029) and rAlt a 1 (27.3% vs. 3.4%, *p* = 0.034) was more common in the EoE group. These findings indicate a higher prevalence of inhalant-related sensitization signals in patients with EoE, consistent with an atopic background enriched for aeroallergen exposure.

Within the lipid transfer protein (LTP) family, sensitization to nArt v 3 was observed exclusively in patients with EoE (18.2%) and was not detected in the CU group (*p* = 0.029). In contrast, sensitization to rVes v 51 was detected only in CU patients (20.7%) and was absent in the EoE cohort (*p* = 0.031). Additional allergen components demonstrated nominal differences in prevalence between groups without reaching statistical significance (Table 5).

Given the number of component-level comparisons performed, the limited cohort size, and the absence of correction for multiple testing, these findings should be interpreted cautiously. Observed differences are intended to highlight potential patterns of sensitization rather than to establish robust component-specific associations. Accordingly, the results presented in this section are descriptive and hypothesis-generating.

### 3.7. Peripheral Blood Eosinophilia

Peripheral blood eosinophilia was evaluated in both study groups using binary classification (presence vs. absence) as well as absolute eosinophil counts. As summarized in Table 6, peripheral eosinophilia was more frequently observed in patients with eosinophilic esophagitis (EoE) than in patients with chronic urticaria (36.4% vs. 3.4%, respectively; *p* = 0.009).

Absolute eosinophil counts were also compared between groups. Due to non-normal distribution of values, results are presented as medians with interquartile ranges. Median eosinophil counts were higher in the EoE group than in the control group (0.30 G/L [IQR: 0.17–0.61] vs. 0.13 G/L [IQR: 0.06–0.23]; *p* < 0.001), as shown in Table 6.

## 4. Discussion

### 4.1. Principal Findings in the Context of Food Ingredient Characterization

The present study provides a descriptive characterization of molecular IgE sensitization patterns in adult patients with eosinophilic esophagitis within a real-world, retrospective cohort. Overall, IgE sensitization to inhalant-related allergen components—most notably PR-10 proteins—represented the most prevalent molecular signal in the EoE group. However, similar sensitization patterns were also observed in the control cohort with chronic urticaria, indicating that this finding reflects a shared cross-reactive atopic background rather than a disease-specific signature.

In contrast, sensitization to selected food-derived allergen components was observed preferentially in patients with eosinophilic esophagitis. Specifically, IgE reactivity to lipid transfer proteins and plant storage proteins was detected only in the EoE cohort and was absent in the control group. While the prevalence of these sensitizations was limited, their selective occurrence suggests a qualitative difference in molecular sensitization patterns between groups rather than a generalized increase in IgE reactivity.

Importantly, these observations should not be interpreted as evidence of causality or as confirmation of disease-driving mechanisms. IgE reactivity in this context reflects sensitization patterns and background exposure rather than a disease-driving immune mechanism. Given the limited sample size, lack of multivariable adjustment, and exploratory study design, the detected differences represent descriptive associations rather than statistically robust or disease-specific effects.

Beyond molecular sensitization profiles, patients with eosinophilic esophagitis exhibited higher rates of peripheral blood eosinophilia and higher absolute eosinophil counts compared with controls. Although peripheral eosinophilia is neither universal nor specific to EoE and does not reliably correlate with disease activity, its coexistence with selective sensitization to structurally stable food allergen components may reflect a broader systemic imprint associated with chronic, food-related mucosal inflammation.

Demographic characteristics of the EoE cohort, including younger age and male predominance, were consistent with established epidemiological patterns of eosinophilic esophagitis. Nevertheless, the lack of age- and sex-matching between study groups introduces the possibility of residual confounding. Differences in allergen exposure, atopic background, or immune maturation related to age or sex may have contributed to the observed sensitization patterns and cannot be fully disentangled from disease-related effects in the present analysis.

Taken together, these findings suggest that, in eosinophilic esophagitis, the relevance of food exposure may be reflected less by the overall burden of IgE sensitization and more by qualitative patterns of sensitization to selected food allergen families. All conclusions drawn from this study remain descriptive and hypothesis-generating and require confirmation in larger, prospectively designed cohorts with standardized clinical and dietary outcome measures.

### 4.2. Inhalant-Driven Sensitization as a Dominant IgE Background

In our cohort, inhalant-related sensitization signals were more frequently detected in patients with eosinophilic esophagitis, with higher prevalence of rBet v 1, rPhl p 5, and rAlt a 1 compared with the control group, alongside a high overall prevalence of PR-10 sensitization (Table 5 and Table 6). These findings are consistent with the concept that, in adult EoE, molecular IgE profiles are often shaped by aeroallergen exposure and cross-reactive pathways rather than by primary sensitization to causative food ingredients. Previous component-resolved studies have similarly demonstrated predominance of birch pollen sensitization with food cross-reactivity in adults with EoE, supporting an inhalant-driven imprint as a common background signal in this disease [26,28].

From a food ingredient perspective, dominance of PR-10 sensitization is best interpreted as a marker of a cross-reactive sensitization landscape, frequently associated with pollen–food syndrome, rather than as an indicator of specific dietary antigens sustaining esophageal inflammation. Although PR-10 sensitization rates vary across populations and may identify subgroups enriched for oral allergy symptoms or broader polysensitization patterns [29,30,31], multiple studies emphasize that molecular IgE sensitization profiles alone have limited utility for identifying trigger foods in eosinophilic esophagitis [26,28]. These observations reinforce the need to distinguish between an inhalant-driven IgE background and food ingredient-related sensitization patterns with potentially greater relevance for chronic, tissue-restricted inflammation.

### 4.3. Structurally Stable Food Allergens as Phenotypic Signals in Food-Driven Eosinophilic Esophagitis

In the present study, sensitization to structurally stable food allergen components was observed preferentially in patients with eosinophilic esophagitis. Lipid transfer proteins were detected only in the EoE cohort, while no LTP sensitization was observed in the control group (*p* = 0.029), and sensitization to plant storage proteins was likewise confined to patients with EoE, albeit without reaching statistical significance. At the molecular level, individual LTP components, including rPru p 3, rCor a 8, and nJug r 3, were identified exclusively in patients with eosinophilic esophagitis (Table 5), contrasting with the broadly distributed inhalant-driven and cross-reactive sensitization background described in Section 4.2.

From a biochemical perspective, lipid transfer proteins represent a class of plant food allergens characterized by high intrinsic structural stability. Their compact tertiary structure, reinforced by multiple conserved disulfide bonds, confers resistance to thermal processing and gastrointestinal proteolysis [32,33,34,35]. As a result, LTP molecules may persist during food processing and gastrointestinal transit, enabling repeated exposure at the mucosal interface. These properties distinguish LTPs from labile allergen families, such as PR-10 proteins, and provide a biologically plausible framework for interpreting their detection in the context of chronic dietary exposure rather than acute IgE-mediated reactions.

Although prior studies on LTPs have predominantly focused on IgE-mediated systemic reactions and anaphylaxis, emerging evidence indicates that immune responses to LTPs may extend beyond classical IgE effector pathways [36,37]. Transcriptomic and immunological analyses have demonstrated alterations in epithelial barrier-related pathways and engagement of non-IgE immune mechanisms in the gastrointestinal tract. However, it is important to emphasize that the present study does not provide functional or mucosal evidence supporting a direct mechanistic role of IgE sensitization or structurally stable food allergens in the pathogenesis of eosinophilic esophagitis.

Accordingly, the selective presence of LTPs and plant storage proteins in patients with eosinophilic esophagitis is best interpreted as an associative finding and a potential immunological marker of sustained food-related exposure rather than as evidence of causative disease drivers. These observations should be considered descriptive and hypothesis-generating. Further studies integrating molecular sensitization profiles with standardized dietary interventions, mucosal immune assessments, and longitudinal clinical outcomes are required to clarify the biological relevance of structurally stable food allergen components in eosinophilic esophagitis.

Accordingly, these findings should be viewed strictly as descriptive signals that warrant further investigation in larger, prospectively designed cohorts.

### 4.4. Molecular Properties of Food Ingredients Beyond Classical IgE-Mediated Allergy

Although IgE sensitization to multiple food and inhalant components was frequently observed in the present cohort, classical IgE-mediated effector reactions did not define the clinical phenotype of eosinophilic esophagitis. Episodes of anaphylaxis were infrequent and occurred with comparable prevalence in the control group, indicating that acute systemic reactions were neither dominant nor specific to EoE. This dissociation between IgE sensitization profiles and clinical expression suggests that IgE reactivity alone is insufficient to account for the chronic, tissue-restricted inflammatory course of the disease [38,39].

Growing evidence indicates that the immunological relevance of food allergens is influenced not only by IgE-binding capacity but also by intrinsic molecular properties of individual food ingredients. Structural stability, resistance to digestion, and susceptibility to processing-induced modification determine whether food-derived proteins persist during gastrointestinal transit and repeatedly interact with the mucosal immune system [38,40]. Many major food allergens belong to protein families characterized by compact folding or disulfide bond stabilization, features that promote survival in the gastrointestinal environment [39,41].

Thermal processing further modulates these properties in an allergen-specific manner. While extensive heating may reduce allergenicity of labile proteins, processing of intrinsically stable allergens can generate structurally modified molecules with enhanced immunogenic potential, for example through glycation and aggregation [40,41,42]. In addition, structurally altered food allergens may interact with intestinal epithelial cells and influence local immune activation, while digestion may reduce IgE-binding epitopes yet preserve T-cell epitopes, potentially shifting immune responses toward sustained, low-grade inflammation rather than acute effector reactions [38,40,43].

Taken together, these observations support the concept that eosinophilic esophagitis represents a disease model in which molecular properties of food ingredients may contribute to chronic immune activation beyond classical IgE-mediated allergy. This framework should be interpreted as hypothesis-generating and underscores the need for immunological approaches that extend beyond serological IgE profiling to better capture mechanisms relevant to chronic, tissue-restricted inflammation [38,39,40].

### 4.5. Diagnostic and Translational Implications of CRD in Food-Driven Diseases

The present study indicates that component-resolved diagnostics (CRD) have limited ability to identify clinically relevant trigger foods in eosinophilic esophagitis. Although molecular IgE sensitization was frequently detected, CRD profiles showed no consistent correlation with foods implicated by dietary interventions, and marked interindividual heterogeneity in the number and composition of sensitized components was observed (Section 3.4). These findings suggest that CRD primarily reflects patterns of sensitization rather than disease-driving mechanisms in EoE.

Previous studies support this interpretation, demonstrating both false-positive and false-negative CRD results in EoE, driven by low specific IgE levels, incomplete panel coverage, and frequent cross-reactivity between inhalant and food allergens [27,44,45]. In adult EoE, birch pollen-associated sensitization is common and may lead to misattribution of plant-derived foods as causative triggers if cross-reactive PR-10 patterns are not adequately recognized [46]. Conversely, allergenic proteins relevant to major EoE triggers, particularly wheat, remain incompletely characterized or absent from current CRD panels, further limiting diagnostic accuracy in this disease context [44,47].

Importantly, CRD findings correlate poorly with clinical outcomes in eosinophilic esophagitis. High rates of molecular sensitization do not reliably predict response to elimination diets, and IgE-targeted therapies have not demonstrated clinical benefit, underscoring the limited pathogenic role of IgE-mediated mechanisms in this disease [27,44]. While selected studies report symptomatic improvement following CRD-guided dietary interventions, the absence of direct comparisons with empiric elimination strategies and the persistence of non-responders highlight the context-dependent and limited predictive value of this approach [45].

From a translational perspective, these limitations reflect the fact that current CRD platforms were developed primarily to assess the risk of acute, IgE-mediated reactions rather than chronic, tissue-restricted inflammation. In eosinophilic esophagitis, CRD may therefore be better positioned as a supportive phenotyping tool rather than a stand-alone guide for dietary decision-making. In this context, the selective detection of structurally stable food allergen components may help identify patient subgroups in whom food-driven antigenic exposure is more prominent, although such applications require prospective validation integrating dietary exposure, clinical response, and biomarkers of chronic inflammation.

### 4.6. Strengths and Limitations

The present study has several important limitations that must be carefully considered when interpreting its findings. Foremost, the small sample size substantially limits statistical power, particularly for component-level analyses, and renders all statistical findings inherently fragile. Given the rarity of eosinophilic esophagitis and the retrospective, single-center design, the study cohort represents all eligible patients with available component-resolved diagnostic data during the study period rather than a statistically powered sample. Consequently, the analyses were not intended to support confirmatory inference.

In addition, no correction for multiple comparisons was applied. In the context of numerous component-level analyses performed on a limited dataset, this increases the risk of false-positive findings. Accordingly, all nominal *p*-values should be interpreted with caution, and observed differences should be regarded as descriptive signals rather than statistically definitive evidence of disease-specific associations.

Another important limitation is the lack of matching between the eosinophilic esophagitis and control groups with respect to age and sex, as well as the absence of multivariable adjustment. Although the demographic distribution of the EoE cohort is consistent with known epidemiological patterns of the disease, residual confounding related to age, sex, atopic background, or allergen exposure cannot be excluded. As a result, the observed differences in molecular sensitization patterns may reflect, at least in part, demographic or exposure-related factors rather than disease-specific effects. In particular, the apparent absence of sensitization to structurally stable food allergen components in the control group should be interpreted cautiously, as false-negative findings cannot be excluded given the limited sample size and the low prevalence of these sensitizations. Accordingly, future studies with larger sample sizes should incorporate multivariable models (e.g., logistic regression adjusted for age and sex) to better disentangle disease-associated sensitization patterns from demographic confounding.

Methodological heterogeneity represents an additional constraint. Two different component-resolved diagnostic platforms (ImmunoCAP ISAC and ALEX2) were used as part of routine clinical practice, with only partial overlap of allergen panels. Although platform distribution did not differ between study groups and platform-stratified sensitivity analyses yielded consistent qualitative findings for the main exploratory endpoint, this heterogeneity further limits the reliability of molecule-specific conclusions, particularly for low-prevalence sensitizations. This limitation is particularly relevant for comparisons of sensitization frequencies at the level of individual allergen components (e.g., Table 5), where differences in platform-specific allergen coverage may influence detection rates. Accordingly, interpretation throughout the manuscript is intentionally focused on allergen families and qualitative sensitization patterns rather than on individual allergen molecules.

A major limitation of the present analysis is the absence of systematically collected clinical correlation data. Standardized information on dietary elimination and reintroduction outcomes, histologic disease severity, endoscopic findings, and treatment response was not uniformly available in the retrospective medical records. Consequently, molecular IgE sensitization patterns could not be linked to clinically relevant endpoints, and no conclusions can be drawn regarding their predictive or therapeutic value.

Finally, it is essential to emphasize that eosinophilic esophagitis is a predominantly non-IgE-mediated disease. The present study does not provide mechanistic insight into disease pathogenesis, nor does it support causal relationships between IgE sensitization to specific allergen components and esophageal inflammation. Instead, the findings should be interpreted strictly as descriptive and hypothesis-generating, highlighting potential phenotypic patterns that warrant validation in larger, prospectively designed cohorts integrating standardized dietary interventions and longitudinal clinical outcomes.

Despite these limitations, the study also has notable strengths. It reflects real-world clinical practice over an extended observation period, includes a clinically relevant allergic comparator group, and applies component-resolved diagnostics to a well-defined adult eosinophilic esophagitis cohort. By explicitly acknowledging its constraints and limiting interpretation accordingly, the present work provides a transparent exploratory framework that may inform future, more definitive investigations into food-related immune phenotypes in eosinophilic esophagitis.

## 5. Conclusions

In this exploratory, retrospective study, we describe molecular IgE sensitization patterns in adult patients with eosinophilic esophagitis using component-resolved diagnostics. Overall, the observed sensitization profiles were dominated by inhalant-related and cross-reactive allergen components, reflecting a shared atopic background rather than disease-specific or IgE-driven mechanisms.

In contrast, sensitization to selected structurally stable food allergen components was detected exclusively in patients with eosinophilic esophagitis. Although the prevalence of these sensitizations was limited, their selective occurrence may represent a phenotypic signal associated with food-driven disease rather than evidence of causative or mechanistic involvement.

These findings do not support the use of component-resolved diagnostics as a stand-alone tool for identifying trigger foods or guiding dietary management in eosinophilic esophagitis. Instead, molecular sensitization profiles should be interpreted cautiously as descriptive markers of immune exposure within a broader clinical context.

Confirmation of these observations requires larger, prospectively designed studies integrating standardized dietary elimination and reintroduction protocols, histologic assessment, and longitudinal clinical outcomes. Accordingly, the present results should be interpreted strictly as descriptive and hypothesis-generating and should not be extrapolated to clinical decision-making.

## Figures and Tables

**Table 1:** Baseline demographic characteristics and type of molecular allergy testing in patients with eosinophilic esophagitis (EoE, *n* = 22) and chronic urticaria (CU, *n* = 29).

Variable	EoE (*n* = 22)	CU (*n* = 29)	*p*-Value
Age, years (median, IQR)	28 (23–35)	39 (29–47)	0.001
Male sex, *n* (%)	13 (59.1)	9 (31.0)	0.053
ISAC, *n* (%)	13 (59.1)	17 (58.6)	1.000
ALEX2, *n* (%)	9 (40.9)	12 (41.4)	1.000

**Table 2:** Prevalence of non-disease-defining atopic comorbidities in patients with eosinophilic esophagitis (EoE, *n* = 22) and chronic urticaria (CU, *n* = 29).

Atopic Comorbidity	EoE (*n* = 22), *n* (%)	CU (*n* = 29), *n* (%)	*p*-Value
Allergic rhinitis	10 (45.5)	8 (27.6)	0.140
Angioedema	2 (9.1)	17 (58.6)	<0.001
Asthma	8 (36.4)	7 (24.1)	0.240
Atopic dermatitis	1 (4.5)	0 (0.0)	0.430
Anaphylaxis	2 (9.1)	2 (6.9)	1.000

**Table 3:** Number of detected molecular allergen components in patients with eosinophilic esophagitis (EoE) and chronic urticaria (CU).

Number of Detected Components	EoE (*n* = 22), *n* (%)	CU (*n* = 29), *n* (%)	*p*-Value
0	5 (22.7)	10 (34.5)	0.536
1–5	3 (13.6)	6 (20.7)	0.714
6–10	2 (9.1)	4 (13.8)	0.688
11–20	7 (31.8)	6 (20.7)	0.518
>20	5 (22.7)	3 (10.3)	0.268

**Table 4:** Sensitization to panallergen families and selected molecular component groups in patients with eosinophilic esophagitis (EoE, *n* = 22) and chronic urticaria (CU, *n* = 29).

Component Group	EoE (*n* = 22), *n* (%)	CU (*n* = 29), *n* (%)	*p*-Value
PR-10	14 (63.6)	11 (37.9)	0.093
LTP	4 (18.2)	0 (0.0)	0.029
Profilins	2 (9.1)	1 (3.4)	0.571
Tropomyosins	1 (4.5)	0 (0.0)	0.431
Storage proteins	3 (13.6)	0 (0.0)	0.074
Parvalbumins	0 (0.0)	0 (0.0)	—
Serum albumins	1 (4.5)	1 (3.4)	1.000
Lipocalins	2 (9.1)	3 (10.3)	1.000
Stable food allergen components	7 (31.8)	0 (0.0)	0.001

**Table 5:** (**A**) Allergen components showing statistically significant differences (*p* < 0.05) between patients with eosinophilic esophagitis and chronic urticaria. (**B**) Allergen components showing nominal differences without statistical significance (*p* ≥ 0.05).

(A)				
Component	Allergen Family	EoE (*n* = 22), *n* (%)	CU (*n* = 29), *n* (%)	*p*-Value
rBet v 1	PR-10 (birch pollen)	14 (63.6)	8 (27.6)	0.021
rPhl p 5	Grass pollen	7 (31.8)	2 (6.9)	0.029
rAlt a 1	Mold (Alternaria)	6 (27.3)	1 (3.4)	0.034
nArt v 3	LTP (mugwort pollen)	4 (18.2)	0 (0.0)	0.029
rVes v 51	Hymenoptera venom	0 (0.0)	6 (20.7)	0.031
(**B**)				
**Component**	**Allergen Family**	**EoE (*n* = 22), *n* (%)**	**CU (*n* = 29), *n* (%)**	***p*-Value**
nPla a 21	PR-10–related pollen	5 (22.7)	1 (3.4)	0.073
nAmb a 1	Ragweed pollen	3 (13.6)	0 (0.0)	0.074
rCor a 8	LTP (hazelnut)	3 (13.6)	0 (0.0)	0.074
nJug r 3	LTP (walnut)	3 (13.6)	0 (0.0)	0.074
rPru p 3	LTP (peach)	3 (13.6)	0 (0.0)	0.074
rPhl p 1	Grass pollen	10 (45.5)	6 (20.7)	0.074
rMal d 1	PR-10 (apple)	12 (54.5)	8 (27.6)	0.082

**Table 6:** Peripheral blood eosinophilia in patients with eosinophilic esophagitis (EoE, *n* = 22) and chronic urticaria (CU, *n* = 29).

Variable	EoE (*n* = 22), *n* (%)	CU (*n* = 29), *n* (%)	*p*-Value
Eosinophilia present	8 (36.4)	1 (3.4)	0.009
Eosinophils, G/L, median (IQR)	0.30 (0.17–0.61)	0.13 (0.06–0.23)	<0.001

## Data Availability

The data presented in this study are available on request from the corresponding author due to privacy and ethical restrictions.
